# Crystalline Insights into Nasal Mucosa Inflammation and Remodeling: Unveiling Role of Galectin-10

**DOI:** 10.3390/biom16010077

**Published:** 2026-01-03

**Authors:** Olga Maria Manna, Velia Malizia, Andrea Perri, Stefania La Grutta, Alberto Fucarino, Domiziana Picone, Mirella Profita, Fabio Bucchieri, Francesca Rappa, Rosalia Gagliardo

**Affiliations:** 1 Pathologic Anatomy Unit, Department of Health Promotion, Mother and Child Care, Internal Medicine and Medical Specialties, University of Palermo, 90127 Palermo, Italy; olgamaria.manna@unipa.it; 2 Euro-Mediterranean Institute of Science and Technology (IEMEST), 90146 Palermo, Italy; 3 Institute of Translational Pharmacology, Italian National Research Council, 90146 Palermo, Italyfrancesca.rappa@unipa.it (F.R.); 4 Department of Earth and Marine Sciences (DiSTeM), University of Palermo, 90123 Palermo, Italy; 5 Department of Theoretical and Applied Sciences, eCampus University, 22060 Novedrate, Italy; 6 Department of Biomedicine, Neuroscience and Advanced Diagnostics (BiND), University of Palermo, 90127 Palermo, Italy

**Keywords:** Galectin-10, Seasonal Allergic Rhinitis (SAR), nasal mucosa, inflammation, remodeling, epithelial–mesenchymal transition (EMT)

## Abstract

Background: Galectin-10 (Gal-10), the main constituent of Charcot–Leyden crystals, is a recognized marker of eosinophilic inflammation, yet its role in nasal mucosal remodeling in Seasonal Allergic Rhinitis (SAR) remains poorly defined. Methods: Gal-10, IL-5, MUC5AC, and IFN-γ were analyzed in Nasal lavage (NL) samples from children with SAR by ELISA. Unsupervised clustering and discriminant analyses were applied. The functional effects of Gal-10 were investigated ex vivo using a 3D epithelial–mesenchymal trophic unit (EMTU) model stimulated with NL containing high, low, or depleted Gal-10 levels. EMT (epithelial–mesenchymal transition) markers (vimentin, E-cadherin, SNAIL1) and MUC5AC secretion were assessed by immunohistochemistry, Western blot, and ELISA. Results: Gal-10 levels in NL positively correlated with IL-5 and MUC5AC and inversely with IFN-γ. Clustering analysis identified distinct SAR endotypes, with Gal-10 showing the highest discriminative power. In the 3D EMTU model, high Gal-10 NL induced increased vimentin and SNAIL1 expression and enhanced MUC5AC secretion, effects attenuated after Gal-10 depletion. Conclusions: Gal-10 is associated with Th2-type inflammation, mucus hypersecretion, and early epithelial–mesenchymal transition in pediatric SAR, supporting its role as a mediator of nasal mucosal remodeling and a potential therapeutic target

## 1. Introduction

Eosinophilic infiltration constitutes a hallmark feature of numerous pathological conditions, including allergic rhinitis and asthma, where the immune system recruits various cell types to affected tissues [1]. Among these, eosinophils and basophils play relevant roles in allergic reactions by releasing highly cytotoxic proteins from their secretory granules, which can induce immunological alterations associated with eosinophil and basophil-mediated inflammation [2].

Seasonal Allergic Rhinitis (SAR) is a heterogeneous inflammatory disease, multi-dimensionally regulated on the Th1, Th2, and Th17 axes. In this context, eosinophilic inflammation and Th2-derived cytokine polarization play a pivotal role along with pro- and anti-inflammatory mediators secreted from serous and mucous glands [3,4,5,6,7]. Recent evidence has shown that several immune-related proteins are differentially expressed in symptomatic patients during the pollen season, although the role of these proteins as potential biomarkers of allergic rhinitis remains to be defined [8,9].

Th2-driven inflammation and eosinophil-related mediators play a central role in the pathogenesis of allergic airway diseases. Type 2 cytokines, including IL-5, profoundly influence epithelial cell behavior, promoting barrier dysfunction, mucus hypersecretion, and tissue remodeling. Increasing evidence indicates that Th2 inflammation contributes to epithelial–mesenchymal transition (EMT). In the nasal mucosa, EMT represents a key mechanism linking chronic inflammation to structural remodeling of the epithelial–mesenchymal trophic unit (EMTU), thereby sustaining disease progression and severity [10,11].

Charcot-Leyden Crystals (CLCs) have frequently been observed at sites of eosinophilic inflammation in diverse biological contexts, including tissues, bodily fluids, and secretions. Therefore, these crystals have been associated with eosinophilic inflammation for several years, raising considerable clinical and scientific interest [12,13].

Initial investigations established that CLCs are composed of crystallized proteins, leading to subsequent inquiries into the biochemical identity of this protein, originally known as CLC-protein (CLC-P), as well as the ability of human eosinophils to store and secrete it [13].

Structural and genomic similarities between CLC-P and the carbohydrate-binding galectin superfamily have led to its reclassification as a galectin, designated as Galectin-10 (Gal-10) [14]. In-depth proteomic analyses have identified CLC-P/Gal-10 as one of the most abundant proteins in eosinophils derived from healthy donors. Over the past few years, several studies demonstrated that substantial quantities of CLC-P/Gal-10 are released during NADPH (reduced nicotinamide adenine dinucleotide phosphate)–dependent eosinophil extracellular trap cell death (EETosis) [15,16].

In various diseases, high levels of Gal-10 protein expression and extracellular CLCs are considered markers of tissue activation and/or eosinophilia [17,18].

In addition to recruiting and activating immune cells, CLCs can induce mucus production by epithelial cells. Overproduction of mucus is a prominent clinical manifestation in many CLC-associated pathologies, including asthma and chronic rhinosinusitis (CRS) [19], where CLC formation promotes tissue damage by recruiting neutrophils and stimulating the production of pro-inflammatory Th1 and Th2 cytokines [19,20].

To date, Gal-10 is considered an effective biomarker of eosinophilic and type-2 inflammation and a potential therapeutic target [19,21]. However, the extracellular crystallization of this protein and its role in the progression of nasal inflammation and mucosa remodeling are not fully understood.

The aim of the present study was to evaluate the potential role of Gal-10 in driving nasal mucosa inflammation, epithelial dysfunction and remodeling in patients with SAR. For this purpose, we first assessed the levels of Gal-10 and its relationship with proinflammatory markers, such as IL-5, MUC5AC and IFN-γ, in nasal lavage (NL) collected from children with SAR. Subsequently, we investigated the potential involvement of Gal-10 in modulating the functional and structural properties of the nasal mucosa using a three-dimensional (3D) ex vivo epithelial–mesenchymal trophic unit (EMTU) model differentiated at the air–liquid interface (ALI). Cultures were exposed to nasal lavage fluid from children with SAR, either containing native or depleted Gal-10, to assess whether intrinsic differences observed in vivo were reproduced under controlled basal conditions.

## 2. Materials and Methods

### 2.1. Patients

The present manuscript reports cross-sectional data from a predefined subgroup of an ongoing clinical study registered on ClinicalTrials.gov (Identifier: NCT03349619) and approved by the local Institutional Ethics Committee (Palermo 1, Palermo, Italy). This subgroup includes pediatric patients who were already under specialist clinical care and had received a confirmed diagnosis of Seasonal Allergic Rhinitis by medical professionals prior to enrollment. Patients selected had complete availability of nasal lavage samples and biomarker data.

The inclusion criteria were: (1) age 6–16 years; (2) diagnosis of AR in the previous year; (3) mono-sensitization to grass pollen, identified by positive skin prick test and specific immunoglobulin E (IgE > 0.70 kU/L). The exclusion criteria were: (1) upper or lower respiratory tract infections (having taken antibacterial therapy in the 4 weeks before the study entry); (2) lifetime history of asthma (doctor diagnosis); (3) use of systemic/topical corticosteroids, systemic/topical decongestants, or antihistamines in the 4 weeks before the study entry; (4) anatomic nasal defects (i.e., septum deviation), or nasal polyps; (5) active smoking.

### 2.2. Nasal Lavage Fluid Collection and Processing

Nasal lavage (NL) was performed by a well-trained physician (VM), as previously described [3]. Subjects were asked to tilt their head back at a 45° angle and close the nasopharynx with the soft palate. NL fluid was obtained by instilling 3 mL of isotonic saline (0.9% NaCl) prewarmed to 37 °C in each patient’s nostril, using a syringe. After 10 s, subjects blew their nose forcefully into a sterile plastic container. The recovered NL fluid volume was comparable to the volume of saline introduced. The average recovery of fluid from NL was approximately 70%. Obtained samples were transferred into conical polypropylene tubes and processed as previously described [22], with minor modifications [3]. Briefly, dithiothreitol (DTT) (Sputolysin, Calbiochem Corp., San Diego, CA, USA), freshly prepared in a 10% dilution with distilled water, was added to the recovered NL fluids in an equivalent volume of 1/10th. After homogenization and centrifugation at 500× *g* for 10 min at 4 °C, the supernatant was recovered and stored at −80 °C for later ELISA assay and 3D culture treatment.

A subgroup of NL samples was selected after characterization and statistical analysis (see above) and subjected to the depletion of Gal-10 protein from fluids by immunoprecipitation, as previously described [23]. All nasal lavage (NL) samples collected from the pediatric SAR cohort were initially quantified for Gal-10 concentration by ELISA. Based on the distribution of Gal-10 values, patients were stratified into low and high Gal-10 groups. This stratification was supported by unsupervised clustering analysis, which consistently identified two biologically distinct profiles characterized by low versus high Gal-10 levels. Sample selection was not performed post hoc and was independent of downstream experimental outcomes. The number of NL samples subjected to immunodepleting was determined by the availability of sufficient lavage volume after diagnostic assays and the need to preserve material for parallel analyses. Briefly, samples were incubated with 2.5 μg of anti-Gal-10 (Abcam, Cambridge, UK) followed by incubation with protein A/G Plus Agarose beads for 1 h at 4 °C. The beads were spun down and the NL supernatants were recovered for subsequent experimental procedures. Gal-10 depletion from nasal lavage samples was validated by re-assessing galectin levels in the supernatant recovered after immunoprecipitation using a quantitative ELISA assay. This analysis demonstrated a depletion efficiency of approximately 80%.

### 2.3. The 3D Epithelial–Mesenchymal Trophic Unit Model

A three-dimensional (3D) model was developed [24,25,26,27] using nasal biopsies obtained from four patients undergoing Functional Endoscopic Sinus Surgery (FESS) at Villa Sofia—Cervello Hospital (Palermo, Italy), with their informed consent and approval from the Ethics Committee of the University of Palermo (Approval code 122/2023—26 January 2023). Patient selection was conducted based on comparable anamnestic characteristics, precisely the absence of acute or infectious diseases and prior corticosteroid therapy within three weeks leading up to the study. Biopsy samples were preserved in Dulbecco’s Modified Eagle Medium (DMEM) supplemented with 10% fetal bovine serum (FBS), penicillin, streptomycin, amphotericin B, and glutamine while maintained on ice. The samples were then transferred to a fresh medium and incubated at 37 °C for 24 h. Subsequently, the samples were sectioned into about 12 fragments each and placed onto 6.5 mm Transwells (BD Biosciences) with approximately 45 μL of Basal Membrane Extract (BME)—Cultrex PathClear, R&D Systems, a composition of specialized proteins derived from the basement membrane and extracellular matrix, including Laminin I, Collagen I, and Collagen IV. Each transwell was positioned in a 24-well plate containing 330 μL of growth medium. The medium consisted of a 1:1 mixture of PneumaCult ALI Maintenance Medium and DMEM supplemented with 10% FBS and 1% penicillin–streptomycin. The growth medium was replaced every two days throughout the culture period, with the cultures maintained in an incubator at 37 °C under a 5% CO_2_ atmosphere.

Upon completion of the culture differentiation, Trans-epithelial Electrical Resistance (TEER) was measured with rod electrodes (EVOM voltmeter, World Precision Instruments, Hitchin, UK). TEER data were integrated with phase-contrast microscope observations to select cultures exhibiting homogeneous growth for subsequent experimental applications.

After a growth and differentiation period of 21 days, the 3D outgrowths were stimulated with NL (20%/vol) for two weeks, leading to the establishment of four distinct experimental groups: samples stimulated with high levels of Gal-10 NL, samples stimulated with low levels of Gal-10 NL, samples stimulated with depleted Gal-10 NL, and control untreated samples. Stimuli were administered to cultures in the basolateral compartment every 48 h for a duration of up to 21 days. The apical surface underwent weekly washing with 250 μL of PBS; this solution, referred to as “apical wash,” was stored at −80 °C for subsequent MUC5AC analysis via ELISA. After the stimulation period, each sample was bisected, with one half utilized for cell lysate extraction, upon which Western blot analysis was conducted to assess SNAIL1 expression (an epithelial–mesenchymal transition marker), while the other half was fixed in 4% paraformaldehyde (PFA) and embedded in paraffin for morphological analysis (hematoxylin-eosin staining) and immunohistochemical assessment of Vimentin and E-cadherin levels of tissue expression.

### 2.4. Preparation of the Cultures for Histology and Immunohistochemistry (IHC) Staining

The outgrowths were fixed by covering the cultured cells with 4% paraformaldehyde at room temperature for 15 to 30 min. Following this, they were rinsed twice with PBS and immersed in 70% ethanol. Inclusion cassettes were prepared by subjecting the samples to increasing ethanol concentrations in 10 min increments, followed by a clearing solvent and melted paraffin. The sections obtained through microtome cutting, with a thickness of 4 to 5 microns, were then immersed in a warm distilled water bath before being placed onto positively charged slides. For the immunohistochemistry analysis, two primary antibodies were selected: vimentin (BIO-Optica-BIO Care Medical, Milan, Italy; Cat. N: PRM 312 AA), a marker for the epithelial–mesenchymal transition, and E-cadherin (BIO-Optica-BIO Care Medical, Milan, Italy, Cat. N: API 3012 AA), a tight-junction marker.

### 2.5. Western Blot Analysis

The outgrowth samples obtained from nasal tissue were subjected to total protein extraction with RIPA lysis buffer and subsequently Western Blot analyses for SNAIL1 (Mouse anti-Human SNAI1 Polyclonal Antibody, Cat. N. MBS642993, MyBioSource, San Diego, CA, USA) and β-actin (Mouse monoclonal antibody, Cat n. ZMS1156, Sigma-Aldrich, St Louis, MO, USA), as previously described [23]. Owing to the limitation in the amount of mucosal sample recovered after bisection of differentiated nasal outgrowths (see Section 2.3), Western Blot was performed in 2 out of 4 samples.

### 2.6. Statistical Analysis

Spearman correlation coefficient was used for assessing correlations between Gal-10 and IL-5, MUC5AC and INFγ measured in the nasal lavage (NL) of children with SAR.

The advanced machine learning technique, Support Vector Clustering (SVC), was used in order to identify patterns and structures within the dataset.

Fisher’s Discriminant Ratio (FDR) was used to evaluate how effectively individual variables distinguished between different groups or clusters in our dataset and how well each variable contributed to differentiating predefined categories.

Ex vivo data were analyzed with ANOVA with Bonferroni correction, permutation ANOVA and Kruskal–Wallis. All the statistical analyses were performed through the statistical software R Studio 4.4. Statistical significance was set at *p*-value < 0.05. One asterisk (*) was used to indicate a *p*-value < 0.01 and two asterisks (**) were used to indicate a *p*-value < 0.001.

## 3. Results

### 3.1. Patients’ Characteristics

Patients’ characteristics are summarized in Table 1.

### 3.2. Determination of Gal-10, IL-5, MUC5AC and IFNγ in Nasal Lavage Fluid of Children with SAR and Cluster Analysis

**Figure 1:**
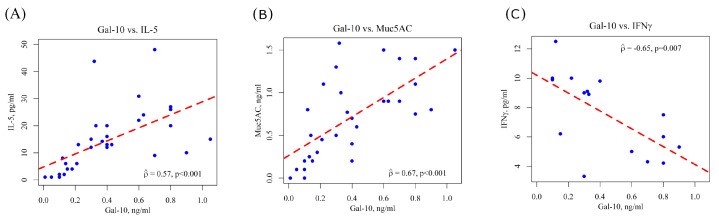
Spearman Correlations between nasal Gal-10 and IL-5 (**A**), MUC5AC (**B**) and IFNγ (**C**) in children with SAR. Correlations were calculated by using the Spearman test.

Gal-10, IL-5, MUC5AC and IFNγ levels in NL were evaluated by ELISA. The results obtained showed that NL levels of Gal-10 positively correlated with IL-5 (ρ = 0.57; *p* < 0.001) and MUC-5AC (ρ = 0.67; *p* < 0.001) concentrations and negatively correlated with IFNγ levels (ρ = −0.65; *p* = 0.007) (Figure 1).

**Figure 2:**
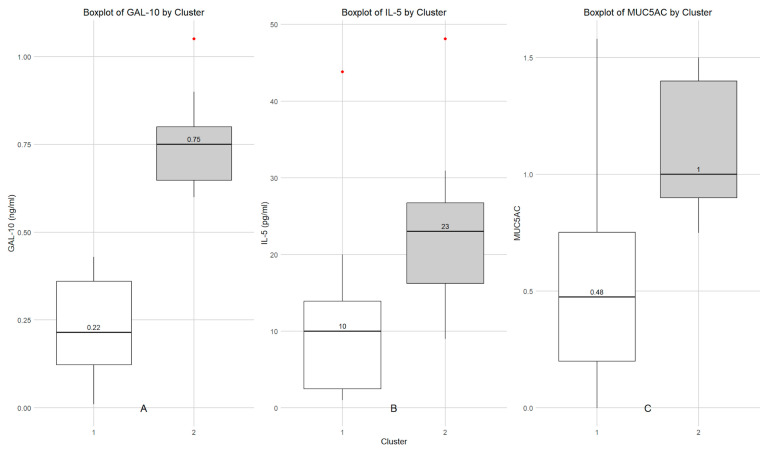
Support Vector Clustering analysis. Boxplot of the distribution of Gal-10 (**A**), IL5 (**B**), and Muc5AC (**C**) nasal levels across different clusters. Each plot represents biomarker variation within its respective clusters, with median values labeled inside the boxes. Outliers are marked in red.

Support Vector Clustering analysis showed two different profiles in SAR patients according to Gal-10, IL-5 and MUCAC measurements (Figure 2). The support vector clustering (SVC) approach was applied in an unsupervised manner, without a priori assumptions regarding the number or nature of patient groups. The algorithm consistently identified two stable clusters defined by the joint distribution of Gal-10, IL-5, and MUC5AC levels. Importantly, cluster separation was not driven by a single biomarker threshold, but emerged from multivariate patterns across these inflammatory mediators, indicating coordinated rather than isolated biomarker behavior.

**Figure 3:**
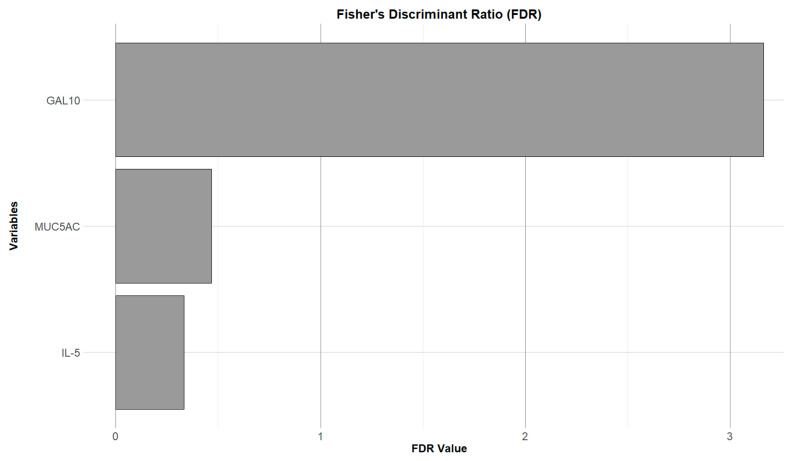
Bar plot of Fisher’s Discriminant Ratio (FDR) values for Gal-10, MUC5AC, and IL-5 in NL samples. The plot highlights Gal-10 as the most discriminant variable among clusters, with the highest FDR value, followed by MUC5AC and IL-5.

To evaluate the effectiveness of individual variables in distinguishing between groups or clusters in patients’ SAR, Fisher’s Discriminant Ratio (FDR) was used. This analysis showed a significantly higher FDR score of Gal-10 (score value: 3.1621) compared to IL-5 (score value: 0.3336) and MUC5AC (score value: 0.4682), indicating its discriminative power in differentiating between SAR patient clusters (Figure 3). This finding indicates that between-cluster variability was predominantly explained by Gal-10 rather than by generic Th2-associated markers such as IL-5 or MUC5AC.

The relevance of this stratification was subsequently explored by testing nasal lavage fluids derived from each cluster in the ex vivo EMTU model, allowing assessment of whether cluster-specific inflammatory profiles translated into differential epithelial remodeling responses.

### 3.3. Morphometric Analysis of 3D EMTU Model

**Figure 4:**
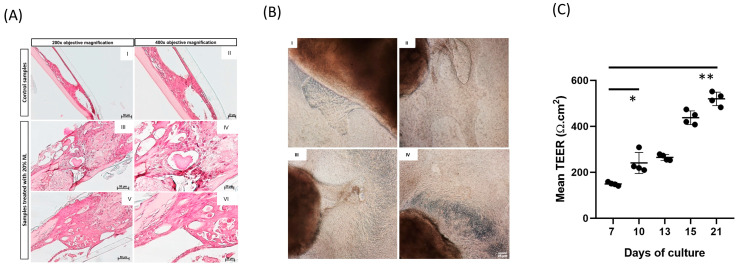
Morphometric and morphological analysis of the 3D EMTU model. (**A**) The panel shows the EE-staining of samples untreated and treated with 20% nasal washes added to the culture medium. (**A_I_**) Control sample (magnification 200×, scale bar 50 µm) (**A_II_**) Control sample (magnification 400× scale bar 20 µm); (**A_III_**–**A_V_**) Treated sample (magnification 200× scale bar 50 µm); (**A_IV_**–**A_VI_**) Treated sample (magnification 400× scale bar 20 µm). In the images of the treated sample, it can be observed that the specimen has appropriately differentiated above the membrane. Additionally, there is a thin layer of connective tissue and an epithelial layer featuring ciliated cells. Notably, a uniform layer of BME, which mimics the extracellular matrix, is also indicated (asterisk). (**B**) The panel depicts four images (200× magnification, scale bar 25 µm) from different biopsies, with the corresponding outgrowths photographed at different times. Specifically, panel (**B_I_**) shows the outgrowth five days post-cultivation, revealing an outgrowth that begins to spread towards the membrane surface’s remaining area (with visible pores) from the biopsy. Panels (**B_II_**,**B_III_**) show the outgrowths one week and ten days after culture, respectively. The outgrowths’ gradual development over numerous layers can be observed. The perceived color differences result from the layering of the newly formed tissue visible under the phase-contrast microscope. (**B_Iv_**) shows the outgrowth two weeks after cultivation, with the tissue well-differentiated and fully confluent. (**C**) TEER measurements (*n* ≥ 4) were carried out during the first 21 days of growth until complete differentiation (calculated by the visible confluence on the membrane surface of the transwells) and the achievement of a homogeneous TEER value for the selected samples. The mean TEER values steadily increased from day 7 until the final time point, reaching a peak at 21 days with a maximum mean TEER value of 346 (SD ± 145). One asterisk (*) was used to indicate a *p*-value < 0.01 and two asterisks (**) were used to indicate a *p*-value < 0.001.

The EMTU model was morphologically characterized using a combination of methods, including monitoring culture growth under a phase-contrast microscope, measuring barrier function (TEER measurements), and histological evaluation via hematoxylin-eosin staining (Figure 4). The cultures originated as an outgrowth of biopsy fragments placed in the center of the transwells. Approximately 10–12 days after culturing, the outgrowth completely covered the transwell membrane, and the culture began to exhibit three-dimensional growth. At this stage, the cells appeared flattened, and BME was still covering the culture. However, by 21 days, the outgrowth gradually degraded the matrix and replaced it with a newly formed extracellular matrix, spontaneously forming a pattern at the air-liquid interface (Figure 4A,B). Histological analysis allowed us to determine the optimal concentration of the NL solution to be used for stimulation of the outgrowths.

We tested three different rates of NL, at 10%, 20%, and 30%, respectively, and observed that rates below 30% NL did not alter the cytoarchitecture of the outgrowths. These results directed our choice toward the use of NL rates of 20% for stimulations.

The fully confluent model displayed a multicellular layer of goblet and ciliated cells, correlating with an increase in TEER that reached a plateau around seven days after reaching the air-liquid interface. In order to assess the barrier function in the EMTU culture model, TEER was measured (*n* ≥ 4) during the first 21 days of growth until complete differentiation (calculated by the visible confluence on the membrane surface of the transwells) and achievement of a homogeneous TEER value for the selected samples. The mean values of TEER showed a consistent increase from day 7 to the final time point, peaking at 21 days with a maximum mean TEER value of 346 (SD ± 145). The plateau of the TEER represented the time from which we obtained homogeneous values in the selected culture samples and thus became the ideal time for starting the apical stimulation with the NL (Figure 4C).

### 3.4. Effects of NL Exposure on EMTU Differentiation of Nasal Outgrowths

The growth and differentiation of 3D nasal mucosal outgrowths were monitored by a phase-contrast microscope (LEICA-DM-IRB, Leica Microsystem S.r.l., Milan, Italy). Nasal mucosal biopsy developed as outgrowths of epithelial and connective tissue, with cells migrating as a coherent sheet to form a multi-layered structure.

Immunoreactivity for e-cadherin and vimentin was evaluated in 3D nasal mucosal outgrowths by immunohistochemistry. The immunomorphological assessment was conducted independently by two observers on two separate occasions to obtain a quantitative analysis of immunopositivity expressed as a percentage. Immunopositivity was evaluated at high-power fields (HPFs, 400× magnification), with assessments repeated across 10 HPFs. The arithmetic mean of the counts was calculated and used for statistical analysis.

**Figure 5:**
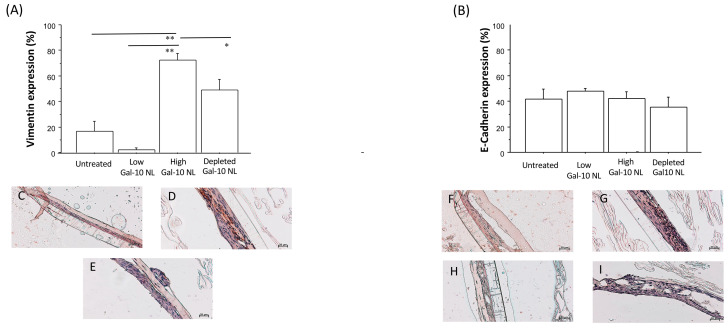
Immunohistochemistry analysis for Vimentin and E-cadherin in 3D nasal outgrowths. Data are expressed as the percentage of positive cells for Vimentin (**A**) and E-cadherin (**B**). Results are expressed as mean and SD; * *p* < 0.01, ** *p* < 0.001. Representative images of immunohistochemistry: (**C**) expression of Vimentin in untreated outgrowths; (**D**) tissue expression Vimentin levels in outgrowths treated with high level Gal-10 NL and (**E**) with NL pretreated for Gal-10 depletion; (**F**) E-cadherin levels of tissue expression of in untreated outgrowths; (**G**) E-cadherin levels of tissue expression in outgrowths treated with high and (**H**) low level Gal-10 NL; and (**I**) with NL pretreated for Gal-10 depletion.

We found an increase in tissue expression of vimentin levels in 3D nasal mucosa outgrowths stimulated with NL, exhibiting high Gal-10 levels in comparison to cultures stimulated with low Gal-10 NL levels and with unstimulated samples. Samples stimulated with NL pre-treated for Gal-10 depletion showed a reduced tissue expression of vimentin levels (Figure 5A). No statistically significant differences were observed in tissue expression of E-cadherin levels between the different experimental conditions (Figure 5B).

**Figure 6:**
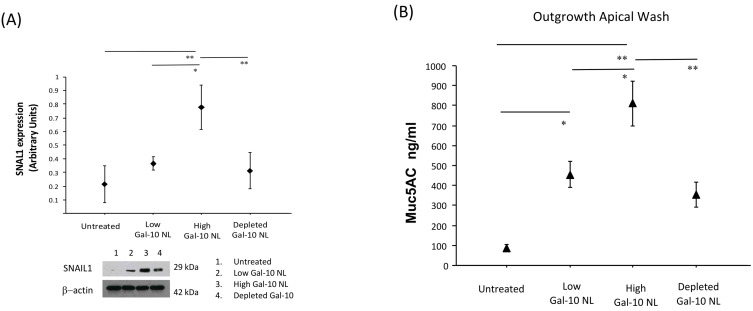
Effects of NL exposure on SNAIL1 expression and MUC5AC secretion in nasal outgrowths. (**A**) SNAIL1 protein expression over 21 days of nasal outgrowth differentiation in the presence or absence of NL with high or low Gal-10 levels. Results are expressed as mean and SD; * *p* < 0.01, ** *p* < 0.001. (**B**) Assessment of MUC5AC secretion in nasal outgrowths (Apical washes) in the presence or absence of NL with high or low Gal-10 levels, after the end of the 7, 14 and 21-day culture period. Results are expressed as means and SDs; * *p* < 0.01, ** *p* < 0.001. Original images can be found in the supplementary materials.

Reconstituted 3D outgrowths of nasal mucosa were also subjected to total protein extraction and subsequent Western Blot analyses for the evaluation of SNAIL1 expression. We found that SNAIL1 protein expression significantly increased in outgrowth samples stimulated with NL containing high Gal-10 protein levels in comparison to samples stimulated with low Gal-10 levels. NL pre-treatment for Gal-10 depletion reduced SNAIL1 protein expression (Figure 6A).

### 3.5. Effects of NL Exposure on MUC5AC Secretion in Apical Washes of Nasal Outgrowths

Muc5AC levels were evaluated in apical washes obtained from 3D nasal mucosa outgrowths. We found that the production of Muc5AC was increased in samples stimulated with NL containing high Gal-10 levels in comparison to cultures stimulated with NL containing low Gal-10 levels and baseline conditions. NL pre-treatment for Gal-10 depletion reduced Muc5AC production (Figure 6B).

Statistical analyses revealed a robust and consistent increase in vimentin expression in nasal outgrowths exposed to NL containing high Gal-10 levels, with all three testing approaches yielding highly significant results. In contrast, E-cadherin expression did not differ significantly across experimental conditions. SNAIL1 showed a trend toward increased expression in the high Gal-10 condition; however, this effect was significant by using ANOVA with Bonferroni correction (Figure 6A) and not consistently supported across non-parametric and permutation-based analyses (Table 2), suggesting weak evidence for a treatment effect, due to sample size limitation. Taken together, these results suggest that high Gal-10 exposure is associated with increased mucin-related output and mesenchymal marker induction, while supporting only a partial or early EMT-like response.

## 4. Discussion

This study provides evidence of Gal-10 involvement in promoting Th2-type inflammation and mucus overproduction in the NL of children with SAR and in de-differentiation and remodeling-related responses. While SAR exhibits a degree of heterogeneity, significant molecular mechanisms underlying this condition are perpetuated by type 2 inflammatory responses and immunity. Charcot–Leyden crystal protein (CLC)/Gal-10, a principal eosinophil-derived component from both cytosolic and granule sources, has been implicated in allergic rhinitis and asthma [20,28]. It is regarded as a potential biomarker for eosinophilic inflammation in respiratory diseases [21], displaying positive predictive significance [29].

A key characteristic of Gal-10 is its tendency to crystallize rapidly and spontaneously under certain conditions, particularly during exocytosis by eosinophils in the extracellular environment, forming Charcot-Leyden Crystals (CLCs). This phenomenon of CLCs is associated with tissue damage commonly observed in respiratory inflammatory disorders, including allergic rhinitis and asthma.

CLCs have been shown to act as immunostimulatory agents capable of triggering the release of pro-inflammatory cytokines (IL-1β, IL-6, TNF-α) and facilitating the recruitment of neutrophils, monocytes, and dendritic cells. However, the mechanisms underlying the increase and crystallization of Gal-10 in CLCs outside the cells, as well as its regulatory role in airway inflammatory processes, remain undefined.

The evaluation of Gal-10 protein levels in the NL of children with SAR highlighted a certain variability in its production within the nasal mucosa of affected individuals, corroborating previous findings on other pro-inflammatory markers in this population (3). Notably, our findings revealed a significant positive correlation between Gal-10 and IL-5 levels, suggesting that type-2 inflammation may promote eosinophil-related Gal-10 production in the nasal mucosa and vice versa, with reciprocal effects on the inflammatory microenvironment. In contrast, we demonstrated a negative correlation between Gal-10 and IFNγ levels in NL, suggesting a lack of association between these markers within the nasal mucosa compartment of the study subjects.

In allergic rhinitis, overproduction of mucus disrupts airway fluid balance, compromising mucociliary clearance and increasing susceptibility to obstruction and infections. Besides their effects on immune cells, CLCs can stimulate mucus production in epithelial cells and can interact with airway mucus via a carbohydrate recognition domain, contributing to the formation of a denser mucus structure that is more difficult to expel by expectoration [30].

Experimental models of allergic rhinitis have demonstrated that the hypersecretion of MUC5AC is mediated by NF-kB and influenced by pro-inflammatory cytokines such as TNF-α, underlining the critical role of MUC5AC as a primary airway mucin [31]. Nonetheless, current literature lacks evidence supporting a direct connection between MUC5AC and Gal-10 in patients with allergic rhinitis. Interestingly, our analysis revealed a positive correlation between nasal levels of Gal-10 and MUC5AC, indicating that Gal-10 activity may serve to promote mucus overproduction and nasal mucosa remodeling in SAR. Moreover, the presence of MUC5AC protein in NL samples of children with SAR implies the potential involvement of the goblet cell phenotype within the nasal mucosa of these patients.

To further characterize the inflammatory and immunological profile of NL in children with SAR, Support Vector Clustering (SVC) analysis was performed on the data obtained, based on the measurements of Gal-10 protein. According to cluster analysis, we identified two distinct subgroups of SAR patients, with low (subgroup 1) or high (subgroup 2) Gal-10 levels in NL fluids. Moreover, the SVC analysis allowed us to identify two different subgroups with low and high IL-5 and MUC5AC values, suggesting the presence of two distinct patient profiles.

To evaluate how effectively individual variables distinguished between different groups or clusters in SAR patients, Fisher’s Discriminant Ratio (FDR) was used. Our results show a significantly higher FDR score of Gal-10 in comparison to IL-5 and MUC5AC, indicating its discriminative power in differentiating between SAR patient clusters and suggesting that the between-cluster variance is large compared to the within-cluster variance.

The limited size of the clinical group analyzed and the absence of a healthy or disease control group represent a limitation of the present study. However, it should be underlined that the integration of in vivo nasal lavage profiling with mechanistic ex vivo experiments using a human three-dimensional epithelial–mesenchymal trophic unit (EMTU) model provides complementary and biologically meaningful evidence that partially compensates for the reduced sample size.

To enhance translational relevance, the adult-derived EMTU cultures were maintained until complete confluence and differentiation, resulting in cytoarchitectural characteristics analogous to those observed in vivo and stimulated with nasal lavage fluids collected from children with SAR, thereby exposing the tissue model to a pediatric disease–specific inflammatory milieu. In this context, we assessed whether varying levels of Gal-10 protein influenced the differentiation processes of nasal mucosa-derived outgrowths.

Moreover, although the initial nasal tissues are of adult origin, our model can accurately replicate the complex architecture of the respiratory epithelium. This is achieved by generating differentiated tissue spontaneously and ex novo from the primary biopsy after approximately 30 days of culture. During this process, a true ‘biological reset’ occurs, enabling the cells to differentiate and reconstitute the mucociliary pseudostratified structure, regardless of the donor’s background. This creates a standardized human platform that mimics the respiratory epithelium’s structural and functional integrity. As a result, our model serves as a neutral and ideal biological system for testing the effects of external stimuli, such as those originating from our pediatric cohort.

TEER was used as a primary quantitative parameter to monitor epithelial integrity and maturation over time, as it is a well-established, non-invasive, and widely accepted indicator of tight junction functionality in air–liquid interface (ALI) and EMTU-based cultures [32]. Importantly, TEER measurements were not used in isolation, but rather integrated with phase-contrast microscopy and histological evaluation (particularly, H&E staining), allowing us to confirm confluence, multilayer organisation, and the establishment of a structured epithelial compartment before experimental stimulation. Under these conditions, TEER defined the time frame during which the model achieved functional stability and uniformity and served as a parameter for comparing crops that had achieved a comparable degree of differentiation. This was essential for ensuring the reproducibility of subsequent Gal-10–dependent effects, rather than providing a comprehensive characterisation of junctional complexity.

The epithelial–mesenchymal transition and signaling mechanisms are crucial in embryonic development and in the response to injury and tissue repair [33,34]. Consistent with this, it is known that the crosstalk between epithelium and mesenchyme plays a crucial role in the pathogenetic mechanisms underlying chronic respiratory diseases [35,36]. In the experiments performed, outgrowths showed adaptive mechanisms both morphologically and molecularly in response to the NL stimulus. To investigate epithelial–mesenchymal transition (EMT) in our ex vivo model, we evaluated the tissue expression of E-cadherin levels, alongside vimentin, an intermediate filament protein commonly associated with mesenchymal cells and recognized as a late marker for advanced stages of EMT. Our results indicated no significant differences in tissue expression of E-cadherin levels between nasal outgrowths cultured with NL showing high or low levels of Gal-10. However, in outgrowth samples stimulated with NL containing high Gal-10 levels, we found a significant increase in tissue expression of vimentin levels when compared to those exposed to NL with lower Gal-10 levels and to unstimulated samples. This suggests that the possible development of EMT is associated with Gal-10 involvement.

The concluding phase of our study involved the analysis of SNAIL1 protein expression and MUC5AC secretion in nasal outgrowths at the end of two weeks of stimulation.

SNAIL1 is a pivotal transcription factor that controls the initiation of EMT and inhibits the activity of the E-cadherin/*CDH1* gene [37,38]. In our experimental model, we found that the samples stimulated with NL containing high Gal-10 levels exhibited an increased SNAIL1 protein expression when compared to those exposed to NL with lower Gal-10 levels and to unstimulated samples, suggesting that Gal-10 may modulate SNAIL1 activity, contributing to EMT-related processes.

Although the increase in SNAIL1 expression seems in contrast with a lack of a significant reduction in E-cadherin in the same samples, our data suggest that SNAIL1 may have a regulatory or initiating contribution to EMT-related molecular mechanisms, rather than a definitive induction of a complete EMT program. Indeed, this latter observation is further supported by the significant increase in tissue expression of vimentin levels observed in nasal outgrowth samples stimulated with NL containing high Gal-10 levels.

Measurements performed in apical washes, obtained from 3D nasal outgrowths stimulated with high Gal-10 NL levels, exhibited increased secretion of MUC5AC in comparison to samples stimulated with depleted or low Gal-10 NL levels and to baseline, suggesting that goblet cell phenotype in nasal mucosa is potentially induced by the presence of Gal-10 protein. These findings suggest a functional link between Gal-10 and the modulation of mucus production in the upper airways and further support the correlation between Gal-10 and MUC5AC demonstrated in NL of children with SAR.

Nevertheless, these findings should be interpreted as exploratory and hypothesis-generating. Ongoing analyses within the full cohort are required to confirm the disease specificity and generalizability of the observed Gal-10–driven pathways.

Overall, our findings revealed a trend in the expression of epithelial–mesenchymal transition markers and mucosa remodeling when considering morphological, functional and molecular aspects. The EMTU model, effectively, mimics the interactive mechanisms between epithelium and mesenchyme. As a future direction, we propose enriching the epithelial–mesenchymal transition marker panel, at various time intervals and broadening the sample cohort employed in the study.

## 5. Conclusions

Taken together, our data indicate that Gal-10 is associated with Th2-type inflammation, mucus overproduction, and EMT-related remodeling signals in the nasal mucosa of children with SAR, potentially predisposing to structural airway dysfunction and progression of respiratory diseases. This latter consideration is particularly noteworthy, although the establishment of a direct causal relationship will require further confirmation in larger cohorts and mechanistic studies.

## Figures and Tables

**Table 1:** Demographic, clinical and biological characteristics of subjects.

	Mean	1st–3rd Quartiles
Sex (N male/female)	22/10	
Age	9.5	9–11
Total IgE	251.43	68–249.5
Eosinophils %	5.217	3.5–5.8
AR (Allergic Rhinitis) Duration (Years)	3.92	1.5–6
Gal-10	0.3981	0.1475–0.6075
MUC5AC	0.7156	0.2875–1.025
IL-5	14.48	5.5–20
IFNγ	7.42	4.83–9.83

**Table 2:** Statistical comparison of EMT markers and mucin-related measurements across nasal outgrowths exposed to nasal lavage (NL) with different Gal-10 levels. Group differences were assessed using, Kruskal–Wallis test, and permutation bias.

Marker	Kruskal–Wallis *p*	Permutation ANOVA *p*
E-cadherin	0.1190	0.1090
Vimentin	0.0018	<10^−4^
Snail1	0.0900	0.0548

## Data Availability

The raw data supporting the conclusions of this article will be made available by the authors upon reasonable request. Due to internal organizational regulations governing the handling of sensitive information, data cannot be openly published and must be shared on request to ensure proper privacy protection and controlled tracking of data access.

## Supplementary Materials

The following supporting information can be downloaded at: https://www.mdpi.com/article/10.3390/biom16010077/s1, Original images of Western Blotting can be found in Supplementary Materials.

## Abbreviations

The following abbreviations are used in this manuscript:

Gal-10	Galectin-10
CLCs	Charcot-Leyden Crystals
SAR	Seasonal Allergic Rhinitis
NL	Nasal lavage
EMTU	epithelial–mesenchymal trophic unit
EMT	epithelial–mesenchymal transition
ALI	Air-Liquid Interface
TEER	trans-epithelial electrical resistance
