# Supplementary figures and images for: Crystalline Insights into Nasal Mucosa Inflammation and Remodeling: Unveiling Role of Galectin-10
Source: Biomolecules. 2026 Jan 3;16(1):77. doi: 10.3390/biom16010077 (PMC12838890; doi:10.3390/biom16010077)

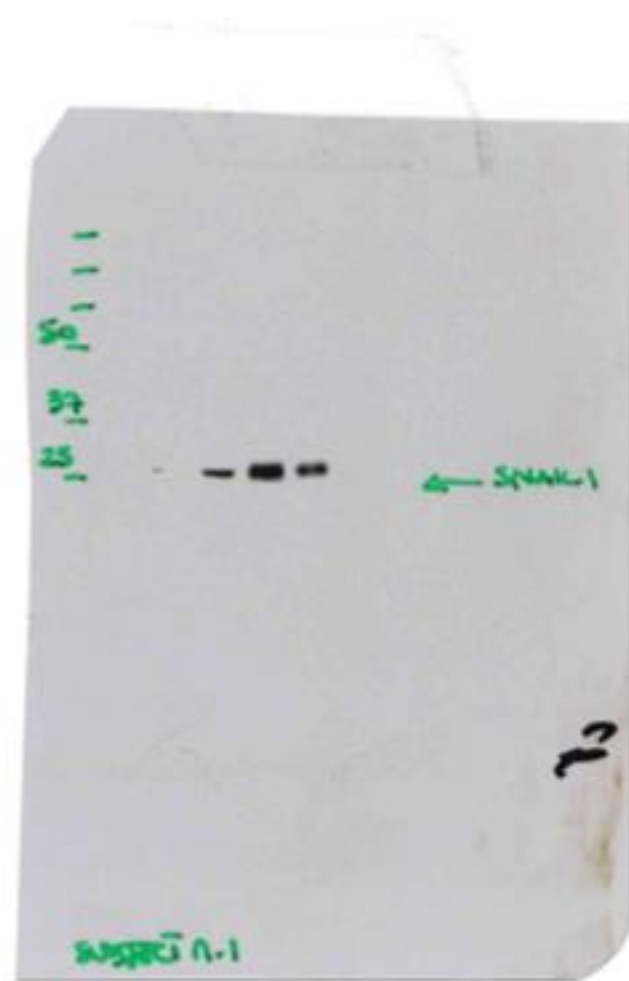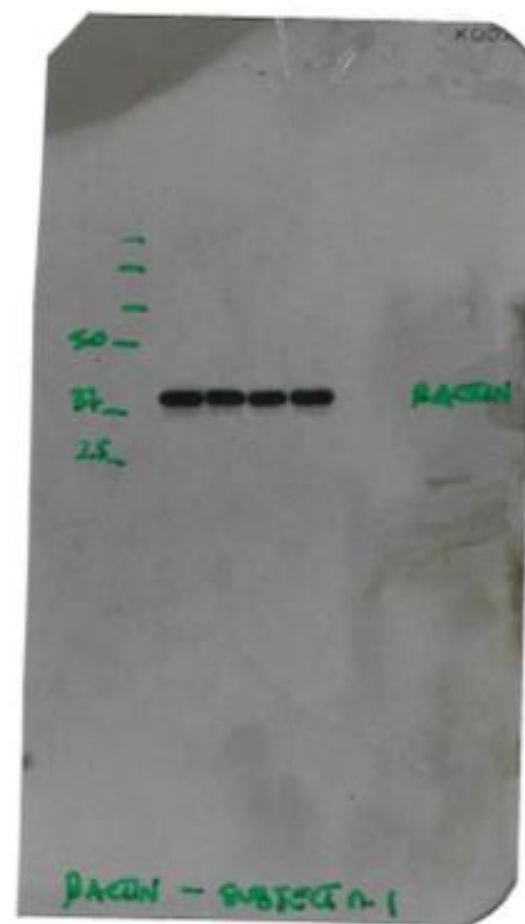

Supplement: Supplementary file 1 [file biomolecules-16-00077-s001.zip › biomolecules-4023372-supplementary.pdf]
